# Hospital Annual Meetings

**Published:** 1919-04-05

**Authors:** 


### HOSPITAL ANNUAL MEETINGS.

### The Royal Free Extension Scheme.

Princess Christian presided at the annual Court of Governors held at the hospital in Gray's Inn Road, London. Mr. Alfred Langton gave particulars of a scheme of extension involving an expenditure of £500,000, which is in contemplation. It was, he said, necessary to have more beds for the general needs of the civilian patients, for treating special diseases, and for the clinical teaching of the students of the London (Royal Free Hospital) School of Medicine for Women. The scheme would entail the erection of a hospital of three floors having eighty beds—the first floor to be devoted to surgery, the second to gynæcology and other special departments, and the third to medicine. Each of these floors would be run by a whole-time professor, assisted by three or four whole-time assistants (there would be a part-time consultant), a pathologist, and a laboratory assistant. Attached to the unit would be theatre rooms, examination rooms, etc. Facilities for research would be a conspicuous feature in the new scheme, and preventive medicine must also be in the forefront of all future study. The hospital would be run on the present lines, having consultant physicians and surgeons and resident staff, and would afford the medical students clinical teaching as at present. If the hospital was not brought up to the modern standard it must cease to exist as a hospital, and the women students, instead of having a "unit" of their own, would be scattered among the men students of other teaching hospitals. Though the cost was great—£500,000—the undertaking was worth it. More than £25,000 has been obtained towards the extension fund.

It was reported that 2,697 in-patients and 26,200 out-patients, whose attendances totalled 120,589, had received treatment during the past year. The total number of beds available for patients was now 200—an increase of twenty-five, due to the incorporation as from September last of the Marlborough Maternity Section, hitherto supported entirely by the Duchess of Marlborough. Income last year amounted to £29,673, and was insufficient to meet the increased expenditure by no less than £8,348. The expenditure, which amounted to £38,022, was nearly £6,000 more than in 1917, and almost £10,000 greater than in 1916. The committee has acquired additional houses in Mecklenburgh Square for the accommodation of nurses, thus setting free a portion of the hospital building, hitherto occupied by the nursing staff, for the use of female patients.

### Charing Cross Hospital.

Ministry of Health Dreaded.

Mr. George Verity, chairman, presiding over the annual meeting, said subscribers had continued to stand by the Charity, which still occupied the proud position of being practically the lowest in cost per bed of any hospital in London. This year was the centenary year of the hospital. During the war the institution had treated over 3,700 soldiers and sailors. When the various Government controls were removed he would be able to view the future with greater feelings of optimism. We never had been, and never would be, a nation to submit to spoon-fed treatment. He really dreaded the new Ministry of Health that was being rushed through. He dreaded its repeating the mistakes and useless expenditure, and, he was almost going to say, the deceits of the Insurance and similar Acts, which, in his opinion, had done more harm than good. He only hoped that the Government was not going to apply, through the Ministry of Health, kindergarten control to the treatment of hospital clinics. It was not the simplest way to strengthen those institutions, the men who were guiding them, and the physicians and surgeons who were bearing the burden of the work in them, nor to get a greater grip on the health of the nation and improve it. If the people concerned would only consult those who knew, instead of evolving new and what they called reformed procedure in dry-as-dust offices, it would be far better for every one.—Mr. Thomas Dewar mentioned that Mr. Verity had collected no less a sum than £350,000 on behalf of the funds of the hospital.

### University College Hospital.

Princess Marie Louise was in the chair at the annual meeting, when it was reported that the income from ordinary sources last year, inclusive of £22,841 from the Government for the maintenance of 1,318 military patients, was £58,203, as against £46,842 in 1917. In addition, extraordinary receipts from legacies amounted to £15,891. Expenditure was £56,031, as compared with £49,186 in 1917, and fell short of the income by over £18,000. In-patients last year numbered 4,978 and out-patients 50,717, with 134,720 attendances. A donation of £2,000 has been received from Mr. Middleton Jameson, in memory of his brother, the late Sir Starr Jameson, to whom a bed has been dedicated in Ward 13. A scheme for the amalgamation of the Royal Ear Hospital, Soho, with the hospital has been completed. A scheme is being developed for the appointment of whole-time teachers of medicine and surgery, together with whole-time assistants.

### Poplar Hospital for Accidents.

The annual Court was held under the presidency of Sir Joseph Broodbank. In-patients to the number of 1,746 were cared for last year, as against 1,908 in 1917, the decrease being partly due to the fewer number of wounded soldiers received—109, as compared with 203. Casualties and out-patients showed the large increase of 6,600, the total being 43,322, of whom 379 were soldiers. The number of beds at the institution is 115. Income amounted last year to £21,594, and was nearly £500 less than in 1917. Expenditure was considerably higher, amounting to £16,889, as against £13,897 a year previously. Two cots at the hospital have been dedicated to the memory of some little children in Poplar, who were killed in an air raid in 1917.

### Mount Vernon Hospital for Consumption.

The Marquis of Zetland presided over the annual Court at Fitzroy Square, W. The majority of the patients, who last year numbered 448, as against 407 in 1917, were, it was stated, ex-soldiers and their dependants. New out-patients to the number of 3,546 with 21,376 attendances, also received attention during 1918, as compared with 6,509 with 16,218 attendances a year previously. Income in the period under review had amounted to £15,910, and was £95 in excess of the expenditure. The pressing need for more beds at Northwood, was especially urged by Mr. Herbert Marnham, chairman of the Committee of Management, who said that the new wing, for which the foundations had already been laid, would, it was hoped, soon be completed and devoted to the treatment of women and children, for whom it was greatly needed.

